# Supplementary material for: A High Temperature Environment Regulates the Olive Oil Biosynthesis Network
Source: Plants (Basel). 2020 Sep 1;9(9):1135. doi: 10.3390/plants9091135 (PMC7569966; doi:10.3390/plants9091135)
Supplement: Supplementary file 1 [file plants-09-01135-s001.zip › supplementary files-Final/plants-891617-supplementary.v3.pdf]

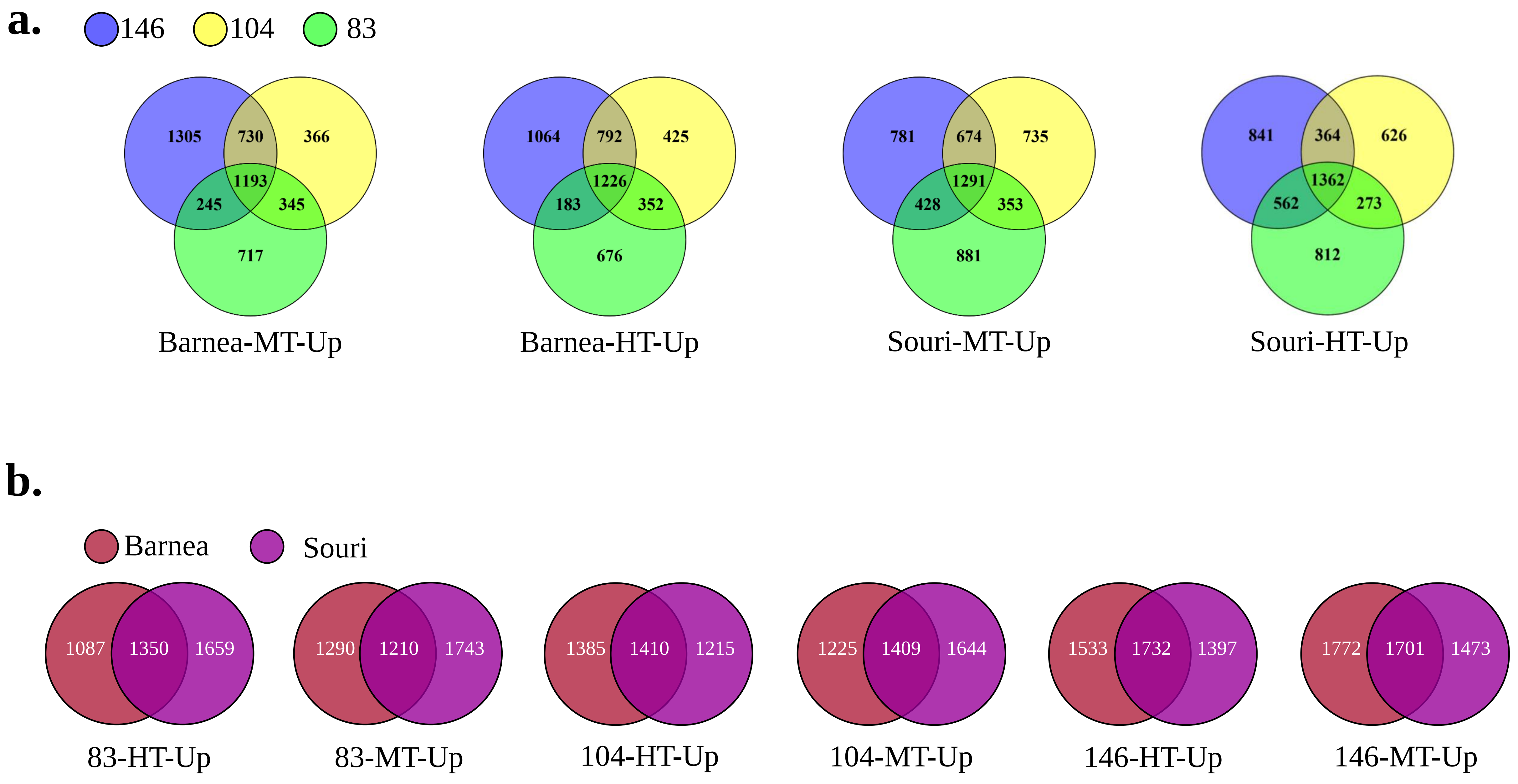
a.
146
104
83
1305
730
366
1193
245
345
717
Barnea-MT-Up
1064
792
425
1226
183
352
676
Barnea-HT-Up
781
674
735
1291
428
353
881
Souri-MT-Up
841
364
626
1362
562
273
812
Souri-HT-Up
b.
Barnea
Souri
1087
1350
1659
83-HT-Up
1290
1210
1743
83-MT-Up
1385
1410
1215
104-HT-Up
1225
1409
1644
104-MT-Up
1533
1732
1397
146-HT-Up
1772
1701
1473
146-MT-Up

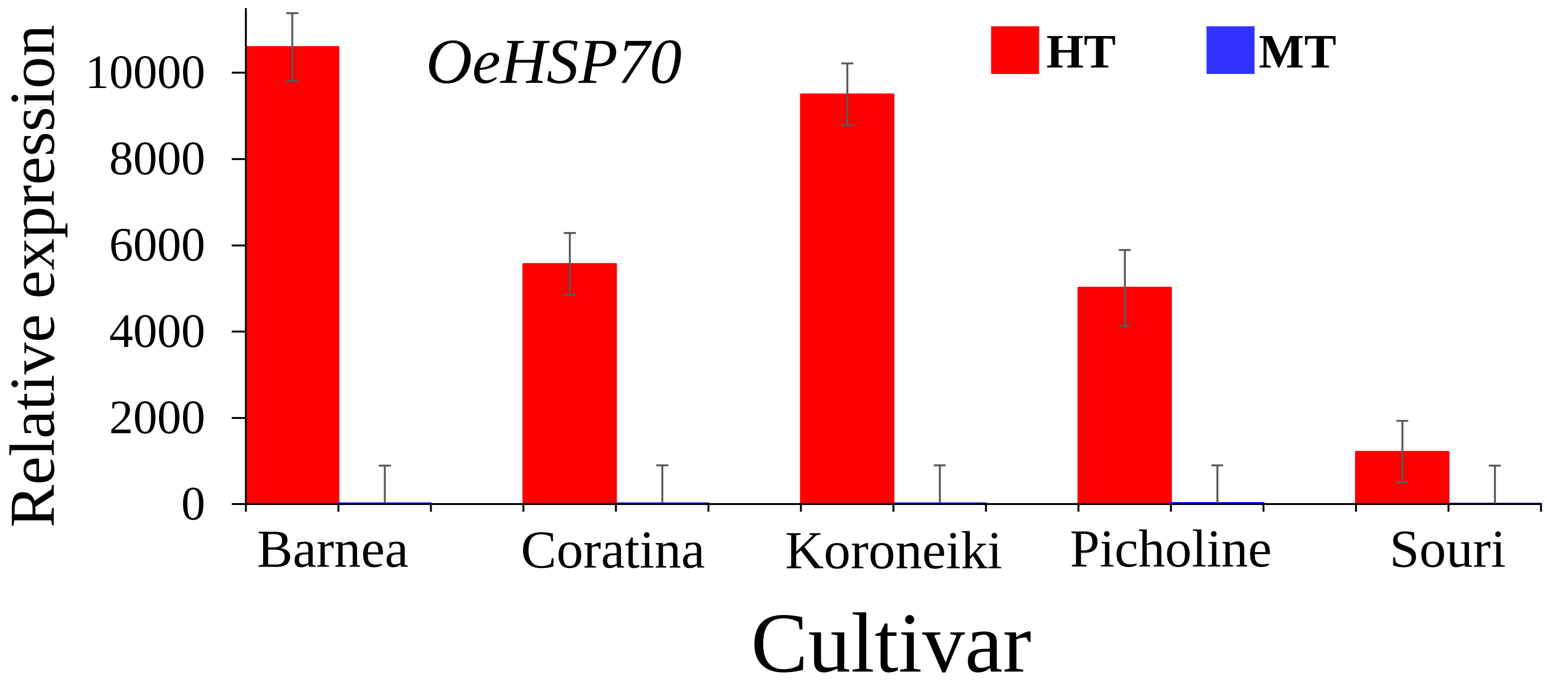

OeHSP70
HT
MT
Relative expression
10000
8000
6000
4000
2000
0
Barnea
Coratina
Koroneiki
Picholine
Souri
Cultivar

Supplementary Table S1

Gene	ID	primer Foreword	primer Reverse
OeHSP70	*OE6A062772*	CAGTTGGCTGAAGTGGATGA	CCAGCACCCTGGTACATCTT
OeACC1	*OE6A049983*	TCACGCATCAGAGAATCCAG	AATCAACGAGGAGCCAATTTT
OeFAD2-1	*OE6A069627*	GGCGACTAAAGCAATCAAGC	CGTCTGGCTCGACATAAAGA
OeActin7	*OE6A117728*	AAGATCAAAGTTGTTGCACCACC	CTTAGAAATCCACATCTGCTGGAAT

Supplementary Table S2

DPA	Cultivar	Environment	#raw-reads	#clean-reads	%mapping
**83**	**Barnea**	**MT**	21,072,968	20,477,528	84.29
		**HT**	19,834,263	19,261,880	83.49
	**Souri**	**MT**	20,062,940	19,482,651	83.39
		**HT**	20,818,192	20,228,179	81.31
**104**	**Barnea**	**MT**	21,285,766	20,414,248	84.42
		**HT**	21,103,525	20,258,222	83.76
	**Souri**	**MT**	19,140,645	18,589,201	83.92
		**HT**	21,422,190	20,817,385	82.39
**146**	**Barnea**	**MT**	24,730,427	23,713,137	84.10
		**HT**	23,577,626	22,615,590	83.89
	**Souri**	**MT**	24,733,850	23,747,805	84.08
		**HT**	23,204,165	22,256,851	82.56
		**Average**	**21,748,879**	**20,988,556**	**83.47**
