# Supplementary material for: A High Temperature Environment Regulates the Olive Oil Biosynthesis Network
Source: Plants (Basel). 2020 Sep 1;9(9):1135. doi: 10.3390/plants9091135 (PMC7569966; doi:10.3390/plants9091135)
Supplement: Supplementary file 1 [file plants-09-01135-s001.zip › supplementary files-Final/Supplementary Figures.docx]

**Supplementary Figures:**


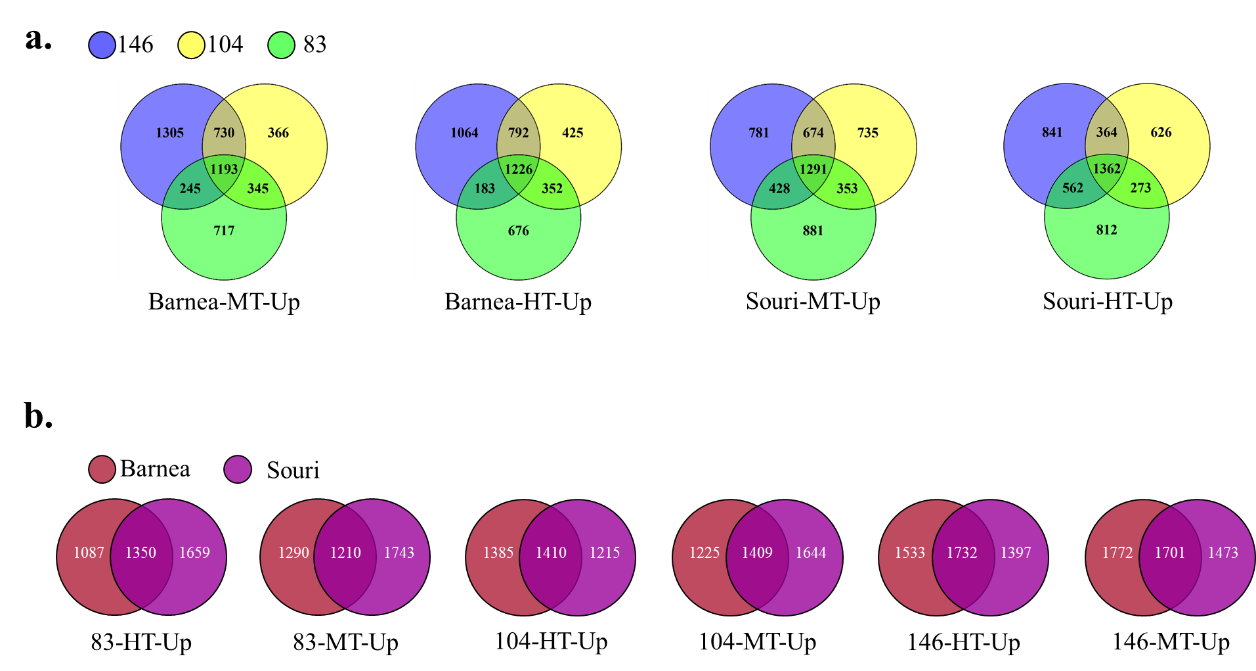


**Figure S1.** Venn diagrams of the significantly regulated genes. Comparison of the regulated gene identities between the different sampled time points in each cultivar and environment (**a**) and between the two cultivars in each time point and environment (**b**). The numbers inside the circles are the number of regulated genes.


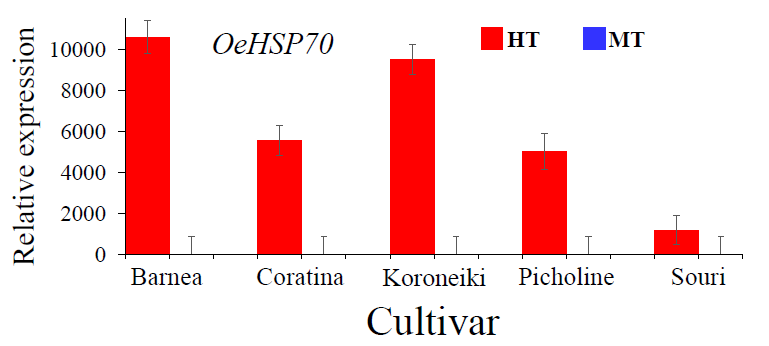


**Figure S2.** *OeHSP70* expression analyzed by RT-PCR at 146 DPA. Relative expression level of *OeHSP70* in the five cultivars 'Barnea', 'Coratina', 'Koroneiki', 'Picholine' and 'Souri', in HT (red columns) and MT (blue columns) environments.
