# Supplementary material for: A High Temperature Environment Regulates the Olive Oil Biosynthesis Network
Source: Plants (Basel). 2020 Sep 1;9(9):1135. doi: 10.3390/plants9091135 (PMC7569966; doi:10.3390/plants9091135)
Supplement: Supplementary file 1 [file plants-09-01135-s001.zip › supplementary files-Final/Supplementary Tables.docx]

**Supplementary Tables:**

**Table S1.** Primers used in the real-time quantitative PCR.

| **Gene** | **ID** | **Foreword Primer** | **Reverse Primer** |
| --- | --- | --- | --- |
| *OeHSP70* | *OE6A062772* | CAGTTGGCTGAAGTGGATGA | CCAGCACCCTGGTACATCTT |
| *OeACC1* | *OE6A049983* | TCACGCATCAGAGAATCCAG | AATCAACGAGGAGCCAATTTT |
| *OeFAD2-1* | *OE6A069627* | GGCGACTAAAGCAATCAAGC | CGTCTGGCTCGACATAAAGA |
| *OeActin7* | *OE6A117728* | AAGATCAAAGTTGTTGCACCACC | CTTAGAAATCCACATCTGCTGGAAT |

**Table S2.** Sequencing results. The left column describe the number of Days Post Anthesis (DPA). #raw-reads are the average number of reads for sequencing lane. #clean-reads are the average number of reads after filtering and % mapping is the percentage of reads mapped to the olive genome.

| **DPA** | **Cultivar** | **Environment** | **#Raw-reads** | **#Clean-reads** | **% Mapping** |
| --- | --- | --- | --- | --- | --- |
| **83** | **Barnea** | **MT** | 21,072,968 | 20,477,528 | 84.29 |
|  |  | **HT** | 19,834,263 | 19,261,880 | 83.49 |
|  | **Souri** | **MT** | 20,062,940 | 19,482,651 | 83.39 |
|  |  | **HT** | 20,818,192 | 20,228,179 | 81.31 |
| **104** | **Barnea** | **MT** | 21,285,766 | 20,414,248 | 84.42 |
|  |  | **HT** | 21,103,525 | 20,258,222 | 83.76 |
|  | **Souri** | **MT** | 19,140,645 | 18,589,201 | 83.92 |
|  |  | **HT** | 21,422,190 | 20,817,385 | 82.39 |
| **146** | **Barnea** | **MT** | 24,730,427 | 23,713,137 | 84.10 |
|  |  | **HT** | 23,577,626 | 22,615,590 | 83.89 |
|  | **Souri** | **MT** | 24,733,850 | 23,747,805 | 84.08 |
|  |  | **HT** | 23,204,165 | 22,256,851 | 82.56 |
|  |  | **Average** | **21,748,879** | **20,988,556** | **83.47** |
